# Ninjurin1 regulates striated muscle growth and differentiation

**DOI:** 10.1371/journal.pone.0216987

**Published:** 2019-05-15

**Authors:** Melanie Kny, Kitti D. Csályi, Kristin Klaeske, Katharina Busch, Alexander M. Meyer, Anne M. Merks, Katrin Darm, Elke Dworatzek, Daniela Fliegner, Istvan Baczko, Vera Regitz-Zagrosek, Christian Butter, Friedrich C. Luft, Daniela Panáková, Jens Fielitz

**Affiliations:** 1 Experimental and Clinical Research Center, Charité-Universitätsmedizin Berlin, Max Delbrück Center for Molecular Medicine in the Helmholtz Association, Berlin, Germany; 2 Electrochemical signaling in development and disease, Max Delbrück Center for Molecular Medicine in the Helmholtz Association, Berlin, Germany; 3 DZHK (German Center for Cardiovascular Research), partner site Greifswald, Greifswald, Germany; 4 University Medicine Greifswald, Department of Internal Medicine B, Greifswald, Germany; 5 Institute of Gender in Medicine, Center for Cardiovascular Research, Charité-Universitätsmedizin Berlin, Berlin, Germany; 6 DZHK (German Center for Cardiovascular Research), partner site Berlin, Berlin, Germany; 7 Department of Pharmacology and Pharmacotherapy, University of Szeged, Szeged, Hungary; 8 Department of Cardiology, Heart Center Brandenburg and Medical University Brandenburg, Bernau, Germany; University of Louisville School of Medicine, UNITED STATES

## Abstract

Chronic pressure overload due to aortic valve stenosis leads to pathological cardiac hypertrophy and heart failure. Hypertrophy is accompanied by an increase in myocyte surface area, which requires a proportional increase in the number of cell-cell and cell-matrix contacts to withstand enhanced workload. In a proteomic analysis we identified nerve injury-induced protein 1 (Ninjurin1), a 16kDa transmembrane cell-surface protein involved in cell adhesion and nerve repair, to be increased in hypertrophic hearts from patients with aortic stenosis. We hypothesised that Ninjurin1 is involved in myocyte hypertrophy. We analyzed cardiac biopsies from aortic-stenosis patients and control patients undergoing elective heart surgery. We studied cardiac hypertrophy in mice after transverse aortic constriction and angiotensin II infusions, and performed mechanistic analyses in cultured myocytes. We assessed the physiological role of *ninjurin1* in zebrafish during heart and skeletal muscle development. Ninjurin1 was increased in hearts of aortic stenosis patients, compared to controls, as well as in hearts from mice with cardiac hypertrophy. Besides the 16kDa Ninjurin1 (Ninjurin1-16) we detected a 24kDa variant of Ninjurin1 (Ninjurin1-24), which was predominantly expressed during myocyte hypertrophy. We disclosed that the higher molecular weight of Ninjurin1-24 was caused by *N*-glycosylation. Ninjurin1-16 was contained in the cytoplasm of myocytes where it colocalized with stress-fibers. In contrast, Ninjurin1-24 was localized at myocyte membranes. Gain and loss-of-function experiments showed that Ninjurin1-24 plays a role in myocyte hypertrophy and myogenic differentiation *in vitro*. Reduced levels of *ninjurin1* impaired cardiac and skeletal muscle development in zebrafish. We conclude that Ninjurin1 contributes to myocyte growth and differentiation, and that these effects are mainly mediated by *N*-glycosylated Ninjurin1-24.

## Introduction

Functional adaptation to physiological and pathological stress situations enables muscle to withstand increased workload [1, 2]. This adaptation involves coordinated development and growth of myofibers and an increase of contractile proteins eventually leading to hypertrophy with an enlarged myocyte surface area. To assure a concise and intact tissue composition in hypertrophy, a proportional increase in the number of cell-cell and cell-matrix contacts mediated by cell-surface proteins is required. Cell-surface proteins are also involved in myogenic differentiation, because they mediate formation of cell-cell contacts between myoblasts, which is a prerequisite for myoblast fusion and formation of multinucleated muscle fibers [3]. Despite their importance in myocyte hypertrophy and myogenesis, only few cell-surface proteins involved in muscle growth and differentiation are described. Examples include myomaker [4], the only muscle-specific cell-adhesion molecule, intercellular adhesion molecule-1 (ICAM-1) [5], and neuronal cell-adhesion molecule (NCAM) [6]. We used a proteomics based approach and identified the cell-adhesion molecule **n**erve **injur**y-**in**duced protein **1** (Ninjurin1), described as two-pass transmembrane cell-surface protein [7], to be increased in the myocardium of patients with severe aortic stenosis, suggesting a role of Ninjurin1 in pathological cardiac hypertrophy. Ninjurin1 is ubiquitously expressed, and conserved from fruit fly to man [8–10]. It was primarily studied in the peripheral nervous system where Ninjurin1 is up-regulated in Schwann cells and neurons after nerve injury and mediates repair processes [7, 8]. By homophilic interaction, Ninjurin1 mediates the contact between macrophages and vascular endothelial cells [11], and enables myeloid cells to migrate through the blood brain barrier in experimental autoimmune encephalomyelitis [12, 13]. Accordingly, germ-line deletion of *Ninjurin1* reduced migration of macrophages into the brain and attenuated disease activity in mice with experimental autoimmune encephalomyelitis [14]. Of note, *N*-glycosylation was shown to be important for *Ninjurin1* homomer assembly [15]. Although, the function of Ninjurin1 in nervous tissue and in myeloid cells is well understood, the biological relevance of Ninjurin1 in heart and skeletal muscle has not been characterized. However, the strong induction of Ninjurin1 as a cell-surface transmembrane protein in the hypertrophic myocardium and its ability to form homomers that mediate cell-cell contacts led us to hypothesize that Ninjurin1 is involved in myocyte hypertrophy and myogenic differentiation. We used heart tissue from patients and mice with pathological cardiac hypertrophy, and performed mechanistic analyses in cultivated myocytes and *zebrafish* to test this hypothesis. We found increased Ninjurin1 protein contents in hypertrophic hearts from patients and mice. Our *in vitro* and *in vivo* experiments demonstrate that Ninjurin1 is involved myocyte growth and differentiation, and that these effects are mainly mediated by *N*-glycosylated Ninjurin1-24. In summary, our data indicate a role of Ninjurin1 in myocyte stress response.

## Methods

### Patients with aortic stenosis and donor hearts

Written informed consent was obtained from all aortic stenosis patients prior to surgery and inclusion in the study, and the study was approved by the ethical committee of Charité Universitätsmedizin Berlin. Myocardial biopsy samples of the left ventricle from nine patients with aortic stenosis were obtained during cardiac surgery. Human non-diseased myocardial tissue was collected from six general organ donors whose hearts were not used for transplantation due to logistical reasons. Sample collection and the experimental protocols were approved by the Ethical Review Board of the Medical Center of the University of Szeged and by the Scientific and Research Ethical Committee of the Medical Scientific Board at the Hungarian Ministry of Health (ETT-TUKEB; No.: 51-57/1997 OEj and 4991-0/2010-1018EKU). The cause of death in general donors included accidents, traumatic cerebral injuries, subarachnoid hemorrhage, subdural hematoma and cerebral aneurism rupture. Tissue samples were frozen in liquid nitrogen immediately after collection and stored at -80°C.

### Animal models of cardiac hypertrophy

All animal procedures were performed in accordance with the guidelines of Charité Universitätsmedizin Berlin as well as Max-Delbrück Center for Molecular Medicine and were approved by the Landesamt für Gesundheit und Soziales (LaGeSo, Berlin, Germany) for the use of laboratory animals (permit numbers: G 0229/11, G 0084/05, IC 114-ZH130) and followed the ‘Principles of Laboratory Animal Care’ (NIH publication no. 86–23, revised 1985) as well as the current version of German Law on the Protection of Animals.

#### Thoracic aortic banding (TAC)

Nine to ten week-old male C57BL/6J mice (purchased from Harlan Winkelmann (Netherlands)) were subjected to pressure overload by 27G thoracic aortic constriction (TAC, n = 5) for 2 weeks as described [16, 17]. The mice were anesthetized with ketamine hydrochloride (80 mg/ml)/xylazine hydrochloride (12 mg/ml) solution administered by intraperitoneal injection at a dose of 1 mg/kg. Animals were placed in supine position under a dissecting stereoscope. Following anesthesia, mice were intubated and artificially ventilated (respirator: Hugo Basile model 7025; FMI). After sternotomy, the aorta and carotid arteries were exposed, and the transverse aorta was constricted by tying a 6–0 silk suture (FST) against a 27-gauge needle. The needle was then removed, leaving a narrowing of 0.4 mm in aortic diameter. A sham procedure in which the thoracic aorta was not banded was performed in littermates for comparison (sham, n = 5). Animals recovered from anesthesia under warming conditions and normal ventilation. As analgesia, carprofen was administered to TAC- and sham-operated mice subcutaneously prior to surgery (10mg/kgKG) and following surgery (every 24 hours for 4 days; 5mg/kgKG).

#### Angiotensin II delivery

Angiotensin (Ang) II (3.0 mg / kg BW / day, human) dissolved in 150 mM NaCl and 1 mM acetic acid was delivered chronically to 12-week-old male C57BL/6J mice (purchased from Charles River, Sulzfeld, Germany) (n = 8) for fourteen days by an implanted osmotic mini-pump (ALZET model 2002) as described previously [17–19]. For control 12-wk-old male C57BL/6J mice (vehicle, n = 5) underwent the same procedure except that the pumps were only loaded with vehicle (150 mM NaCl, 1 mM acetic acid). All mice were anesthetized with isoflurane. As analgesia, carprofen was administered once to all mice by subcutaneous injection (5mg/kgKG) prior to surgery.

All animals were housed under standard conditions with a 12 hour light-dark cycle and controlled temperature (21°C) regime. Water and food were provided ad libitum. Environmental enrichment (shelter and nesting material) was provided to all animals throughout the experiments. Post-operatively the health status of all animals was monitored at least once daily in order to assess animal health and well-being. The criteria used to judge animal health and well-being were based on a predefined checklist and included, beside others, the assessment of animal behavior, fur texture, respiration, changes in movement, apathy, injuries, pain, and changes in body weight. At the end of the respective experiment mice were sacrificed by cervical dislocation; hearts were isolated, and heart weight, body weight, and tibia length were measured.

For detailed information about zebrafish experiments, BD Pharmingen PowerBlot, cell culture, isolation of neonatal rat ventricular myocytes, transfection of cDNA expression plasmids and small interfering RNA (siRNA), cycloheximide chase assay, tunicamycin treatment, protein extraction and Western blot analysis, biochemical fractionation of cells, RNA isolation, cDNA synthesis, quantitative RT-PCR (qRT-PCR), immunocytochemistry and immunohistology, please refer to the S1 File.

### Statistics

Data are presented as mean ± standard deviation (SD) unless otherwise stated. A non-parametric test, the Mann-Whitney test, was performed to analyze group differences for patient samples. Student’s t-tests and one-way ANOVA analyses were used for animal experiments, PCR data and cell culture experiments. *P* < 0.05 was considered as statistically significant.

## Results

### Ninjurin1 is increased in hearts of patients with severe aortic stenosis

**Fig 1:**
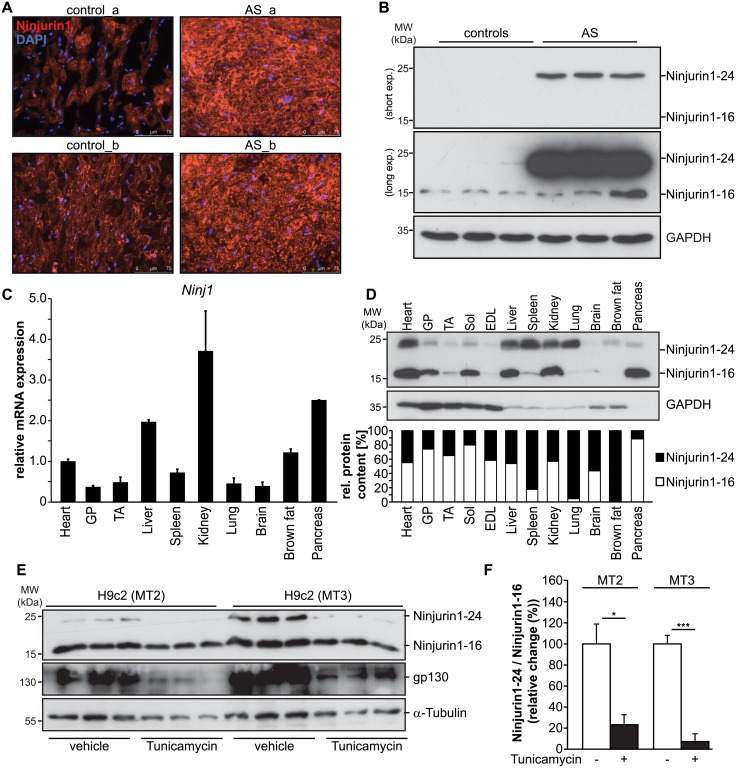
Ninjurin1 is increased in hypertrophic left ventricle of patients with severe aortic stenosis. **(A)** Immunofluorescent staining of cryosections from biopsy specimen of two AS (AS_a, AS_b) and two donor (control_a, control_b) hearts with anti-Ninjurin1 as primary antibody and Alexa Fluor 555 conjugated secondary antibody. Nuclei were stained with DAPI (blue). MW, molecular weight; kDa, kilo Dalton. Scale bar, 75 μm. **(B)** Western blots of proteins isolated from left ventricular tissue of patients with severe aortic stenosis (AS) undergoing elective aortic valve replacement surgery (*n* = 3) and donor hearts (*n* = 3) using anti-Ninjurin1 antibody. GAPDH was used as loading control. The Ninjurin1 isoforms at 16kDa (Ninjurin1-16) and 24kDa (Ninjurin1-24) are differentially expressed therefore the membranes underwent short and long exposure, indicated by short exp. and long exp., respectively. **(C)** Quantitative RT-PCR analysis of *Ninjurin1* (*Ninj1*) in different mouse tissues obtained from untreated C57BL/6J mice as indicated (*n* = 3). *18S ribosomal RNA* expression was used as reference. Data are presented as mean ± SD. **(D)** Representative immunoblot of proteins isolated from different mouse tissues using anti-Ninjurin1 antibody. GAPDH was used as loading control. The Ninjurin1 isoforms at 16kDa (Ninjurin1-16) and 24kDa (Ninjurin1-24) are indicated. Graph: Densitometric analysis of (B). The total Ninjurin1 content (Ninjurin1-16 plus Ninjurin1-24) per tissue was set as 100% and the relative amount of Ninjurin1-16 and Ninjurin1-24, respectively, was related to that. GP indicates *gastrocnemius / plantaris*; TA, *tibialis anterior*; Sol, *soleus*; EDL, *extensor digitorum longus*. (**E**) Western blots of proteins isolated from 2 days (MT2) or 3 days (MT3) differentiated H9c2 cells, treated with 1μg/ml Tunicamycin or vehicle (1 mg/ml dimethylsulfoxide) for 48 h, as indicated. Detection of Ninjurin1 and glycoprotein 130 (gp130) using specific antibodies. Tubulin was used as loading control. (**F**) Densitometric analysis of (E); data are presented as mean ± SD. * *P* = 0.05; *** *P* < 0.001. A two-tailed, unpaired Student’s t-test was used to calculate the *P* values.

To identify proteins regulated in left ventricular hypertrophy (LVH) due to severe aortic stenosis (AS) in humans, proteins extracted from left ventricular biopsy samples of patients undergoing elective aortic valve replacement surgery (n = 9) and from donor hearts (n = 6) were subjected to the BD Pharmingen PowerBlot system and analyzed for differences in protein contents. Patient characteristics are shown in S1 Table. The transmembrane cell adhesion molecule Ninjurin1 was highly expressed in the myocardium of AS patients but not in control samples. Immunohistochemistry with anti-Ninjurin1 antibody on cryosections from left ventricular biopsies of AS patients and donors confirmed that Ninjurin1 was increased in AS compared to donor myocardium (Fig 1A). Western blot experiments on proteins isolated from representative biopsies of the left ventricle of AS patients (n = 3) and donors (n = 3) with anti-Ninjurin1 antibody verified the results from the PowerBlot analysis (Fig 1B). Western blot analysis from a larger cohort of AS patients (n = 9) and donors (n = 6) confirmed these findings (S1 Fig). A Ninjurin1 specific signal at 24kDa (Ninjurin1-24) was detected in AS samples only but not in controls, whereas the 16kDa Ninjurin1 variant (Ninjurin1-16) was expressed at low levels in both groups (Fig 1B). To investigate the expression pattern of Ninjurin1 and to determine the tissue distribution of both Ninjurin1 variants we performed qRT-PCR and Western blot analysis in heart and skeletal muscle samples, and compared it to non-muscle tissue. We found that *Ninjurin1* mRNA was ubiquitously expressed and that its expression in muscle is comparable to non-muscle tissue (Fig 1C). Likewise, Western blot analysis of protein lysates from different mouse tissues showed that Ninjurin1 protein was ubiquitously expressed. However, expression levels and the ratio of Ninjurin1-16 to Ninjurin1-24 differed between the tissues investigated, as also shown by densitometrical analysis (Fig 1D). Since, Ninjurin1 is a transmembrane cell-surface protein [7] and *N*-glycosylation is a hallmark of cell membrane proteins [20] together with a recent report that Ninjurin1 is *N*-glycosylated [15] we reasoned that the molecular weight difference between Ninjurin1-16 and Ninjurin1-24 was due to its *N*-glycosylation. To test this hypothesis we investigated *N*-glycosylation of Ninjurin1 in H9c2 cells. H9c2 cells are an immortalized cell line originally derived from embryonic rat ventricular tissue [21] that shows a cardiac phenotype and are used to investigate mechanisms of cardiomyocyte stress response [22, 23]. Because NRVM and H9c2 cells were shown to respond similar to hypertrophic stimuli [24–28] we used both models in our study. We treated H9c2 cells, which were differentiated for two and three days (MT2, MT3), respectively, with the *N*-glycosylation inhibitor tunicamycin. Western blot analysis and subsequent densitometry showed that these cells contained both Ninjurin1-16 and Ninjurin1-24. Tunicamycin abolished the formation of Ninjurin1-24 (Fig 1E and 1F), indicating that the observed molecular weight difference between Ninjurin1-16 and Ninjurin1-24 was caused by *N*-glycosylation of Ninjurin1. As a control, we used tunicamycin to inhibit *N*-glycosylation of the cell surface receptor glycoprotein 130 (gp130) (Fig 1E).

**Fig 2:**
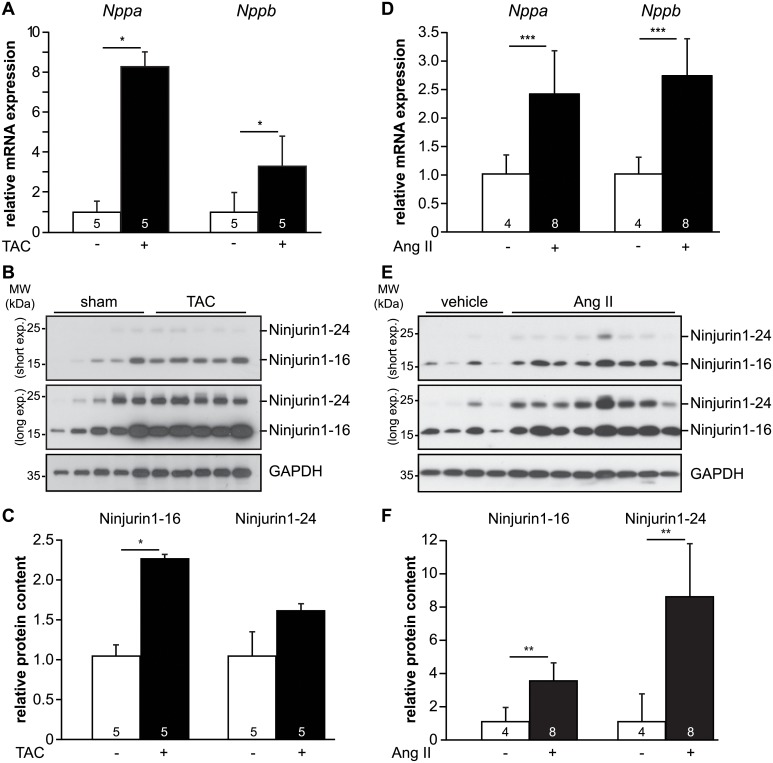
Ninjurin1 expression increases in the left ventricle of mice with pathological left ventricular hypertrophy. (**A**) Quantitative RT-PCR analysis of atrial (*Nppa*) and B-type natriuretic peptide (*Nppb*) on RNA from left ventricular tissue of 9- to 10-wk-old male mice subjected to two weeks of transverse aortic constriction (TAC) (*n* = 5) or sham surgery (*n* = 5). *Glyceraldehyde-3 phosphate dehydrogenase* (*GAPDH*) expression was used as reference. Data are presented as mean ± SD. * *P* < 0.05. **(B)** Immunoblots of proteins isolated from left ventricles of mice subjected to sham (*n* = 5) or TAC (*n* = 5) surgery using anti-Ninjurin1 antibody. GAPDH was used as loading control. The Ninjurin1 isoforms at 16kDa (Ninjurin1-16) and 24kDa (Ninjurin1-24) are differentially expressed, therefore the membranes underwent short (short exp.) and long exposure (long exp.), respectively. (**C**) Densitometric analysis of (B); data are presented as mean ± SEM. * *P* < 0.05. **(D)** Quantitative RT-PCR analysis of *Nppa* and *Nppb* on RNA from left ventricular tissue of 12-wk-old male mice subjected to chronic Angiotensin II (Ang II, *n* = 8) or vehicle (*n* = 4) infusion, respectively, via osmotic mini-pumps for two weeks. *GAPDH* expression was used as reference. Data are presented as mean ± SD. *** *P* < 0.001. **(E)** Immunoblots of proteins isolated from left ventricles of mice subjected to vehicle (*n* = 4) or Ang II (*n* = 8) treatment using anti-Ninjurin1 antibody. GAPDH was used as loading control. (**F**) Densitometric analysis of (E); data are presented as mean ± SEM. ** *P* < 0.01. A two-tailed, unpaired Student’s t-test was used to calculate the *P* values.

To investigate if increased Ninjurin1 expression is a general feature for LVH and if this phenomenon is conserved throughout species, we analyzed the hearts of two standard mouse models of pathological LVH. First, transverse-aortic constriction (TAC, n = 5) and sham (n = 5) operations were performed in wild type mice, and hearts were analyzed two weeks after surgery. As expected, TAC induced LVH as shown by an increase in heart-weight-to-tibia length ratio and increased thickness of the interventricular septum and left ventricular posterior wall during diastole measured by transthoracic echocardiography (S2 Table). Likewise, qRT-PCR on RNA isolated from left ventricular tissue showed an increased atrial natriuretic peptide (*Nppa*) and B-type natriuretic peptide (*Nppb*) gene expression in TAC compared to sham mice (Fig 2A). Western blot analysis and subsequent densitometry showed an increased Ninjurin1 protein content (Ninjurin1-16 and Ninjurin1-24) in hearts of TAC (n = 5) compared to sham (n = 5) animals (Fig 2B and 2C). We also tested Ninjurin1 regulation in response to continuous administration of Angiotensin II (Ang II) via osmotic mini-pumps to mice that also causes LVH. As expected, two weeks of Ang II treatment resulted in LVH, as demonstrated by an increase in heart-weight-to-tibia length ratio (S3 Table), and increased left ventricular *Nppa* and *Nppb* gene expression (Fig 2D). Western blot analysis and subsequent densitometry showed that both Ninjurin1 variants were increased in hearts of Ang II (n = 8) treated mice compared to vehicle treated controls (n = 4) (Fig 2E and 2F). In summary, upregulation of Ninjurin1 accompanies pathological cardiac hypertrophy in both AS patients and animal models of pathological cardiac hypertrophy.

### Reduction of Ninjurin1 inhibits cardiomyocyte hypertrophy *in vitro*

**Fig 3:**
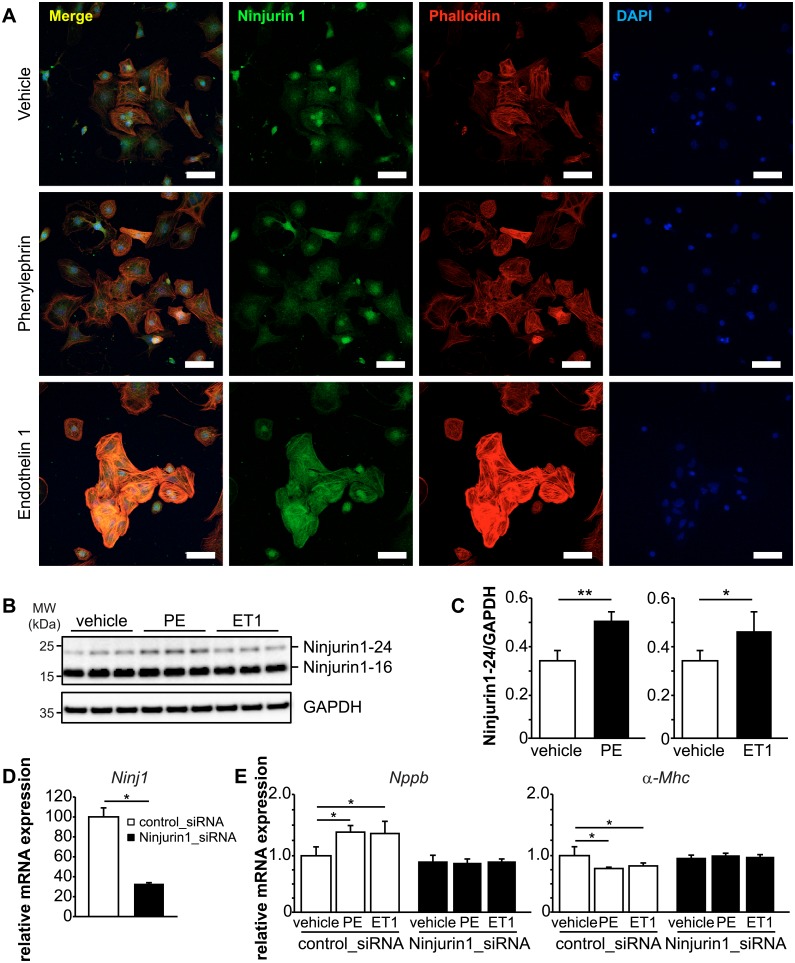
Knockdown of Ninjurin1 by siRNA impairs cardiomyocyte hypertrophy *in vitro*. **(A)** Immunofluorescent staining of NRVM treated with vehicle, phenylephrine (100 μM) and endothelin 1 (50 nM) with anti-Ninjurin1 as primary antibody and Alexa Fluor 488 conjugated secondary antibody (green). Nuclei were stained with DAPI (blue). Scale bar, 75 μm. (**B**) Immunoblots (left) and densitometric analysis (right) of proteins isolated from vehicle, phenylephrine (100 μM) and endothelin 1 (50 nM) treated NRVM using anti-Ninjurin1 antibody. GAPDH was used as loading control. The 16kDa (Ninjurin1-16) and 24kDa (Ninjurin1-24) Ninjurin1 isoforms are indicated. (**C**) Densitometric analysis of the Ninjurin1-24 signal from (B). (**D, E**) NRVM were transfected with control siRNA (control_siRNA) (*n* = 3) or siRNA targeting Ninjurin1 (Ninjurin1_siRNA) (*n* = 3). (**D**) Quantitative RT-PCR analysis of Ninjurin1 (*Ninj1*). *GAPDH* expression was used as reference. Data are presented as mean ± SD. * *P* < 0.05. (**E**) Quantitative RT-PCR analysis of *Nppb* and alpha-myosin heavy chain 1 (*α-Mhc*). *GAPDH* expression was used as reference. Data are presented as mean ± SD. * *P* < 0.05, ** *P* < 0.01, *** *P* < 0.001.

We next investigated if Ninjurin1 is contained in cardiomyocytes and if it plays a role in cardiomyocyte hypertrophy. Immunocytochemistry confirmed that endogenous Ninjurin1 protein was contained in NRVM (Fig 3A). Treatment of NRVM with the hypertrophy inducing compounds phenylephrine (100 μM) and endothelin 1 (50 nM) increased the amount of Ninjurin1 protein in NRVM. Western blot analyses and densitometry revealed that phenylephrine and endothelin 1 treatment caused an increase of Ninjurin1-24 but not Ninjurin1-16 (Fig 3B and 3C). To investigate if Ninjurin1 is important for cardiomyocyte hypertrophy we used siRNA to reduce Ninjurin1 expression in NRVM and compared them to cells treated with scrambled siRNA controls (Fig 3D). As expected and as shown by qRT-PCR, phenylephrin and endothelin 1 caused an increase in *Nppb* and a decrease in *α-Mhc* mRNA expression in control siRNA treated NRVM (Fig 3E). However, knockdown of Ninjurin1 decreased phenylephrine- and endothelin 1-induced *Nppb* mRNA expression compared to controls (Fig 3E). Likewise, Ninjurin1 knockdown abolished phenylephrine- and endothelin 1-mediated reduction of *a-Mhc* mRNA expression (Fig 3E).

**Fig 4:**
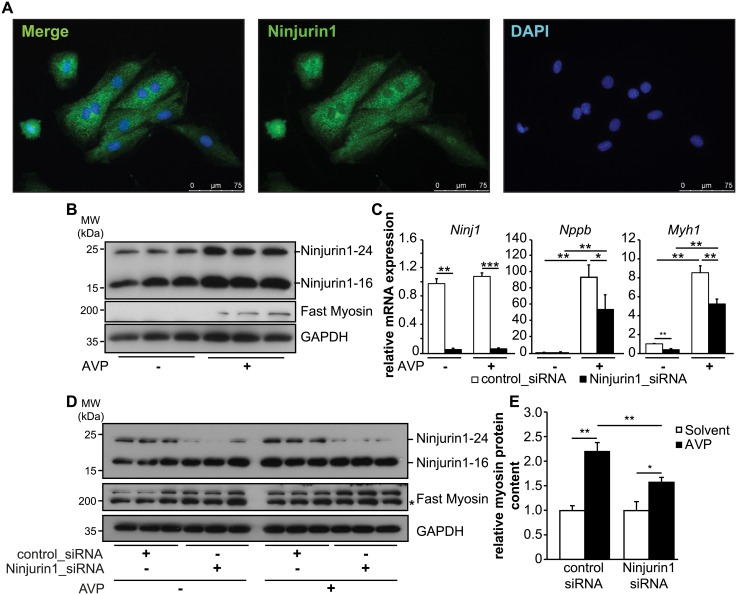
Knockdown of Ninjurin1 by siRNA leads to impaired cardiomyocyte hypertrophy *in vitro*. **(A)** Immunofluorescent staining of H9c2 myocytes with anti-Ninjurin1 as primary antibody and Alexa Fluor 488 conjugated secondary antibody (green). Nuclei were stained with DAPI (blue). Scale bar, 75 μm. (**B**) Hypertrophy of 7 days differentiated H9c2 myotubes was induced by arginine-vasopressin (AVP) treatment for 24 hours. Non-treated cells were used as control. Immunoblots of proteins isolated from H9c2 myotubes (as indicated) using anti-Ninjurin1 and anti-fast myosin antibody. GAPDH was used as loading control. The 16kDa (Ninjurin1-16) and 24kDa (Ninjurin1-24) Ninjurin1 isoforms are indicated. **(C-E)** 7 days differentiated H9c2 myotubes were transfected with control siRNA (control_siRNA) (*n* = 6) or siRNA targeting Ninjurin1 (Ninjurin1_siRNA) (*n* = 6). 48 hours after transfection hypertrophy was induced by AVP treatment for 24 hours (*n* = 3). Non-treated cells were used as control (*n* = 3). (**C**) Quantitative RT-PCR analysis of Ninjurin1 (*Ninj1*), *Nppb* and Myosin heavy chain 1 (*Myh1*). *GAPDH* expression was used as reference. Data are presented as mean ± SD. * *P* < 0.05, ** *P* < 0.01, *** *P* < 0.001(**D**) Immunoblots of proteins isolated from H9c2 myotubes (as indicated) using anti-Ninjurin1 and anti-fast myosin antibody. GAPDH was used as loading control. The 16kDa (Ninjurin1-16) and 24kDa (Ninjurin1-24) Ninjurin1 isoforms are indicated. (**E**) Densitometric analysis of fast myosin protein content from (D). Data are presented as mean ± SD. * *P* < 0.05, ** *P* < 0.01. MW, molecular weight; kDa, kilo Dalton. A two-tailed, unpaired Student’s t-test was used to calculate the *P* values.

To substantiate our findings we investigated the role of Ninjurin1 in H9c2 cells. Using immunocytochemistry, we found that Ninjurin1 was contained in H9c2 cells (Fig 4A). We next treated H9c2 myocytes with arginine-vasopressin (AVP), known to induce hypertrophy of these cells [29]. Western blot analysis showed that AVP increased Ninjurin1-16 and Ninjurin1-24, as well as fast Myosin protein in H9c2 cells (Fig 4B). To investigate if Ninjurin1 is important for hypertrophy, we used siRNA to reduce Ninjurin1 expression in H9c2 cells and compared them to cells treated with scrambled siRNA controls. As shown by qRT-PCR knockdown of Ninjurin1 decreased AVP induced *Nppb* and *Myh1* mRNA expression compared to controls (Fig 4C). Western blotting and its densitometry showed that reduction of Ninjurin1 decreased the ability of AVP to increase the myosin protein content (Fig 4D and 4E). In summary, our data indicate that Ninjurin1 plays a role in cardiomyocyte hypertrophy *in vitro*.

### Ninjurin1 expression is increased during myogenic differentiation

**Fig 5:**
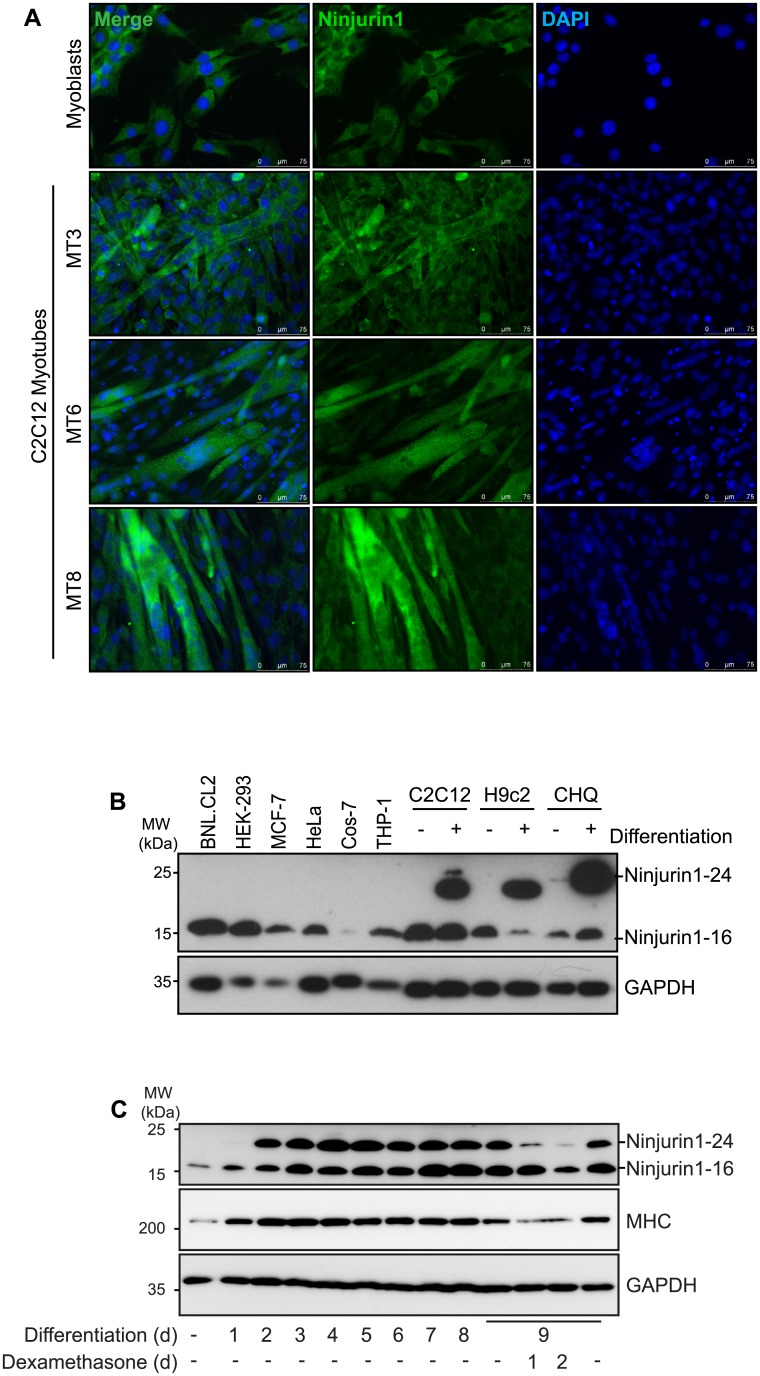
Ninjurin1 increases during myogenic differentiation in skeletal myocytes. **(A)** Immunofluorescent staining of C2C12 myoblasts, and for 3, 6 and 8 days differentiated myotubes, as indicated (MT3, MT6, MT8), with anti-Ninjurin1 as primary antibody and Alexa Fluor 488 conjugated secondary antibody (green). Nuclei were stained with DAPI (blue). Scale bar, 75 μm. (**B**) Western blots of proteins isolated from different non-myocyte (BNLCL2, HEK-293, MCF-7, HeLa, Cos-7, THP-1) and myocyte (C2C12, H9c2, CHQ) cell lines (as indicated) using anti-Ninjurin1 antibody. The myocyte cell lines C2C12, H9c2 and CHQ were analyzed as undifferentiated myoblasts (-) and differentiated myotubes (+). GAPDH was used as loading control. The 16kDa (Ninjurin1-16) and 24kDa (Ninjurin1-24) Ninjurin1 variants are indicated. **(C)** Western blots of proteins isolated from C2C12 myoblasts and for different time points (as indicated) differentiated myotubes using anti-Ninjurin1 antibody. To induce atrophy 9 days differentiated C2C12 myotubes were treated with Dexamethasone (10 μM) for 1 and 2 days, as indicated. GAPDH was used as loading control. The 16kDa (Ninjurin1-16) and 24kDa (Ninjurin1-24) Ninjurin1 isoforms are indicated. MHC indicates myosin heavy chain.

Our data showed that Ninjurin1 was not only expressed in the heart but also in skeletal muscle (Fig 1C and 1D). They also showed that skeletal muscle contained Ninjurin1-16 as well as Ninjurin1-24 (Fig 1C and 1D). Importantly, Ninjurin1-24 which increases in LVH was exclusively expressed in differentiated skeletal myotubes (Fig 5). Therefore, we hypothesized that Ninjurin1 does not only play a role in cardiomyocyte hypertrophy but also in skeletal myocyte growth and differentiation. Using immunocytochemistry, we confirmed that endogenous Ninjurin1 protein was contained in C2C12 myoblasts (Fig 5A) and that its immunoreactivity steadily increased during differentiation of C2C12 myoblasts to myotubes (Fig 5A). Western blot analysis revealed that Ninjurin1-16 was contained in myoblasts as well as in myotubes as well as in non-myocyte cell lines (Fig 5B). In contrast, *N*-glycosylated Ninjurin1 (Ninjurin1-24) was exclusively detectable in differentiated myotubes (Fig 5B). To investigate the kinetics of Ninjurin1s *N*-glycosylation during myogenic differentiation we performed Western blot analyses of lysates from myoblasts and myotubes at different stages of differentiation in C2C12 cells. *N*-glycosylated Ninjurin1 (Ninjurin1-24) was first detected after 48 hours of differentiation, and its expression gradually increased during differentiation until day 5 when it reached a steady state (Fig 5C). In contrast, Ninjurin1-16 was already present in myoblasts and its amount did not change during differentiation. To test if myotube atrophy has an effect on Ninjurin1 we treated differentiated C2C12 myotubes with the glucocorticosteroid dexamethasone (Dexa), an established atrophy inducer. Western blot analysis showed a reduction particularly of the Ninjurin1-24 protein content in C2C12 myotubes after 24 hours and 48 hours of Dexa-treatment (Fig 5C). In summary, our data implicate that *N*-glycosylated Ninjurin1 (Ninjurin1-24) plays a role in myogenic differentiation.

We further investigated the subcellular localization of Ninjurin1-16 and Ninjurin1-24. By immunocytochemistry of myoblasts, which do not contain Ninjurin1-24, we found that Ninjurin1-16 was co-localized with actinin-containing structures that are important for migration (S2A Fig). Neither Ninjurin1-16 nor Ninjurin1-24 was colocalized with GAPDH, which was used as a marker for soluble cytosolic proteins (S2B Fig). Because *N*-glycosylation of proteins can affect their intracellular trafficking, we performed biochemical fractionation of myoblasts and myotubes to investigate if Ninjurin1-16 and Ninjurin1-24 localize to different intracellular compartments. Indeed, both Ninjurin1 variants were located to different subcellular compartments; whereas, Ninjurin1-24 was enriched in membrane containing fractions, Ninjurin1-16 co-precipitated with vesicles (S2C Fig). In summary, the different expression patterns, differentiation dependent *N*-glycosylation and divergent cellular distribution point towards specific functions of Ninjurin1-16 and Ninjurin1-24 in myocytes.

### Ninjurin1 contributes to myogenic differentiation *in vitro*

**Fig 6:**
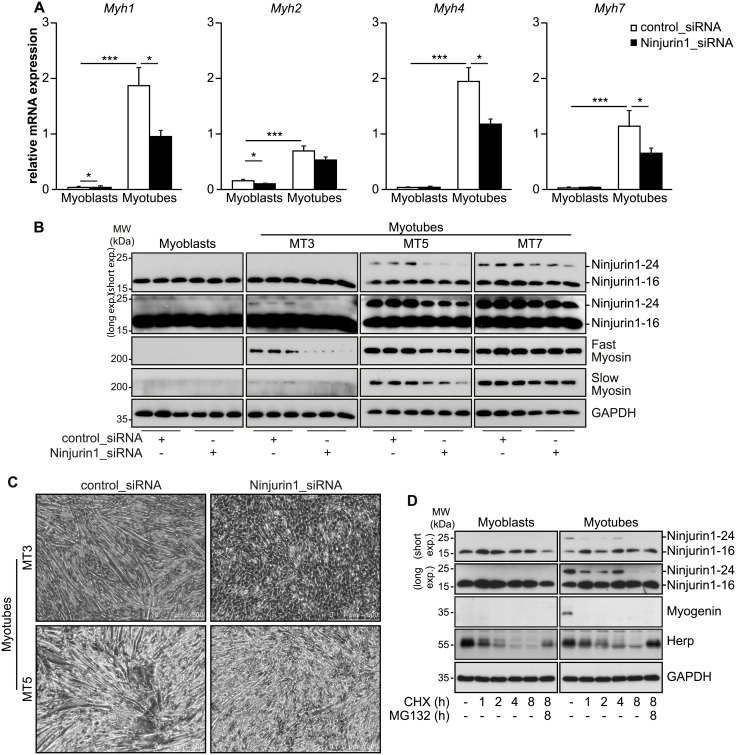
Knockdown of Ninjurin1 impairs myogenic differentiation. C2C12 myoblasts were transfected with control siRNA (control_siRNA) or siRNA targeting Ninjurin1 (Ninjurin1_siRNA). Differentiation was initiated 24 hours after transfection. **(A)** RNA was isolated from C2C12 myoblasts and after 7 days of differentiation (MT7). Quantitative RT-PCR analysis of myosin heavy chain (*Myh) 1*, *2*, *4 and 7* is shown. *Glyceraldehyde-3 phosphate dehydrogenase* (*GAPDH*) expression was used as reference. Data are presented as mean ± SD. A two-tailed, unpaired Student’s t-test was used to calculate the *P* values. * *P* < 0.05, ** *P* < 0.01, *** *P* < 0.001. *n* = 3. **(B)** Western blots of proteins isolated from C2C12 myoblasts, and for 3, 5 and 7 days differentiated myotubes, as indicated (MT3, MT5, MT7), with anti-Ninjurin1, slow myosin and anti-fast myosin antibody (as indicated). GAPDH was used as loading control. The Ninjurin1 isoforms at 16kDa (Ninjurin1-16) and 24kDa (Ninjurin1-24) are differentially expressed, therefore the membranes underwent short and long exposure, indicated by short exp. and long exp., respectively. The 16kDa (Ninjurin1-16) and 24kDa (Ninjurin1-24) Ninjurin1 isoforms are indicated. **(C)** Microscopy pictures of differentiated C2C12 myocytes at day 3 (MT3) and 5 (MT5) of differentiation following transfection with control siRNA (control_siRNA) and siRNA targeting Ninjurin1 (Ninjurin1_siRNA), respectively. Scale bar, 500 μm. (**D**) Western blots of proteins isolated from C2C12 myoblasts, and 7 days differentiated myotubes, treated with cycloheximide (CHX) and MG132 for different time points, as indicated, with anti-Ninjurin1, Myogenin and Herp antibody (as indicated). GAPDH was used as loading control.

Since Ninjurin1 expression increased during myogenic differentiation, we reasoned that Ninjurin1 is involved in this process. We used siRNA to reduce endogenous Ninjurin1 in myoblasts and investigated its effect on differentiation. We used qRT-PCR and Western blot analysis to quantify Myh gene expression and protein content as late markers for myogenic differentiation [30]. qRT-PCR showed the expected increase in *Myh1*, *2*, *4* and *7* gene expression after seven days of differentiation. The increase in *Myh1*, *4* and *7* gene expression was significantly diminished by Ninjurin1 siRNA (Fig 6A). Western blot analysis showed that knockdown of Ninjurin1 led to a decrease of both fast-myosin and slow-myosin protein after three and five days of differentiation (Fig 6B). Microscopical analysis showed that knockdown of Ninjurin1 abolished myotube formation after three and five days of differentiation compared to controls (Fig 6C). Because Western blot analysis showed that Ninjurin1 siRNA treatment only diminished Ninjurin1-24 but not Ninjurin1-16 protein after 3 days, 5 days and 7 days of differentiation (Fig 6B) we hypothesized that the half-life of Ninjurin1-16 is longer than that of Ninjurin1-24. Therefore, we tested the stability of the Ninjurin1 variants using cycloheximide decay assays and used the proteasome inhibitor MG132 to determine if protein degradation was proteasome dependent. The assays were controlled by determination of Herp and Myogenin protein contents, which are degraded in a proteasome dependent and independent manner, respectively. We found that *N*-glycosylated Ninjurin1 (Ninjurin1-24) was degraded within 8 hours and that its degradation was not dependent on the proteasome. In contrast, Ninjurin1-16 was not degraded throughout the duration of the experiment indicating that Ninjurin1-16 is highly stable (Fig 6D).

Because knockdown of Ninjurin1 inhibited myogenic differentiation, we reasoned that elevated Ninjurin1 levels would accelerate this process. We therefore overexpressed FLAG-tagged Ninjurin1 in C2C12 myoblasts, differentiated them for 3 days, 5 days and 7 days and investigated markers of myogenic differentiation. Western blot analysis using anti-FLAG antibody confirmed effective Ninjurin1 overexpression (S3A Fig). As expected, all tested *Myh* genes significantly increased after three, five and seven days of differentiation compared to myoblasts (S3B Fig). Importantly, compared to empty vector control Ninjurin1 overexpression resulted in an increased *Myh1* expression throughout differentiation. Ninjurin1 overexpression also increased *Myh2* expression, at days 3 and 7 of differentiation, *Myh4* expression, at days 3 and 5 of differentiation, and *Myh7* expression at differentiation day 3 (S3B Fig). Western blotting showed that Ninjurin1 overexpression increased fast-myosin protein contents at day 5 and 7 of differentiation. However, Ninjurin1 overexpression had no effect on slow myosin protein content (S3A Fig). In concert, our loss- and gain-of-function data show that Ninjurin1 contributes to myogenic differentiation.

### Ninjurin1 depletion in zebrafish results in impaired heart and skeletal muscle development

**Fig 7:**
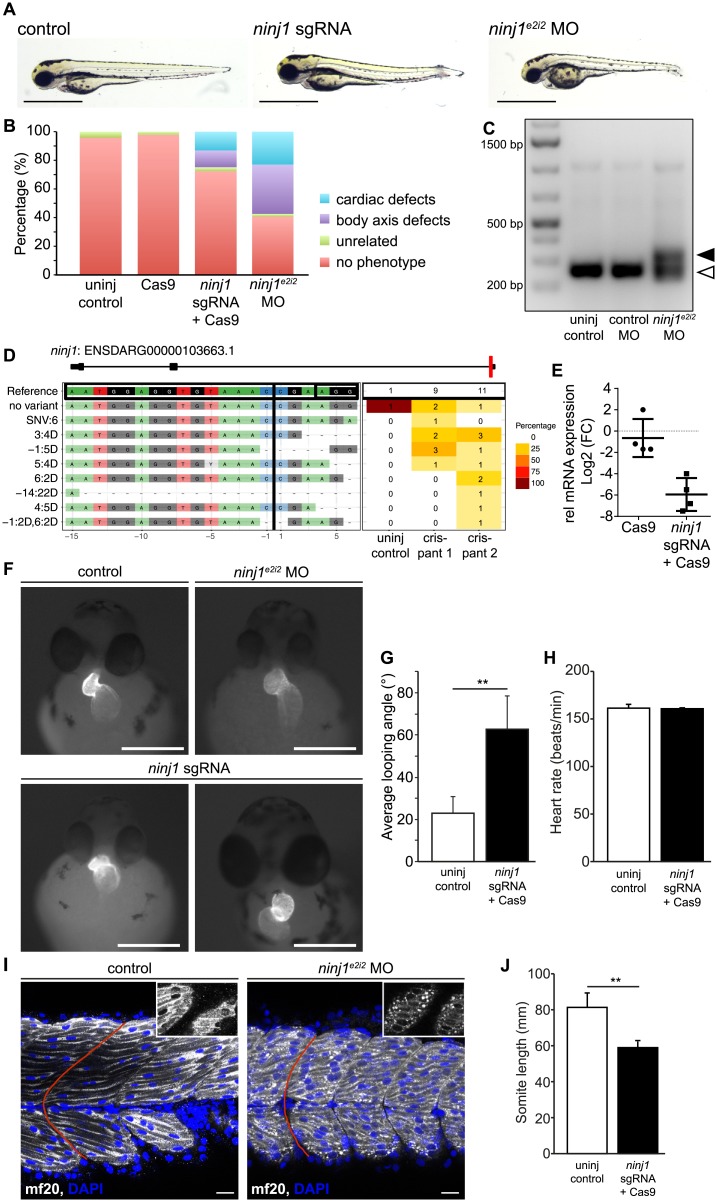
Ninjurin1 deficiency impairs zebrafish heart and skeletal muscle development. Zebrafish (wild type or transgenic *Tg(myl7*:*EGFP)*^twu34^) embryos were injected with ribonucleoprotein complex of Cas9 and sgRNA targeting exon 1 of *ninjurin1* (*ninj1* sgRNA) or morpholino targeting exon2/intron2 junction of *ninjurin1* (*ninj1*^*e2i2*^ MO) at one cell stage and compared to uninjected control. In all panels, embryos were analysed at 48–50 hours post fertilization (hpf). (**A**) Representative images of uninjected controls and *ninjurin1*-deficient embryos showing cardiac and body axis defects. Scale bar, 1 mm. (**B**) The relative occurrence of the cardiac phenotypes is depicted. (**C**) qRT-PCR of *ninj1* cDNA from uninjected control, control morpholino injected (control MO), and *ninj*^e2i2^ MO injected embryos at 50 hpf. In control MO injected embryos, the size of the *ninj1* amplified fragment is 254 bp (empty arrowhead), whereas it is approximately 350 bp in *ninj1* morphants, due to defective splicing of intron 2 (full arrowhead). (**D**) Panel plot showing allele variations in *ninj1 sgRNA*-Cas9-injected embryos compared to the WT *ninj1* allele (no variant) (ENSDARG000001036663.1). (**E**) Relative mRNA expression of the *ninj1* gene in uninjected, Cas9, and *ninj1 sgRNA*-Cas9-injected embryos by qRT-PCR and normalized to *eef1α1l* mRNA expression. Data represent the log_2_ fold change of *ninj1*. Cas9 to uninjected control, ns, *P* = 0.0686, *ninj1 sgRNA-Cas9*-injected embryos to uninjected controls, **, *P* = 0.0025. (**F**) Representative images of *Tg(myl7*:*EGFP)*^twu34^ showing defects in cardiac looping and dextrocardia in *ninj1*-deficient embryos. Scale bar, 1 mm. (**G**) Quantification of the looping angle, *ninj1 sgRNA*-Cas9-injected embryos to uninjected control, **, *P* = 0.0012. (**H**) Heart rate remained unchanged in *ninj1*-deficient embryos; *ninj1 sgRNA*-Cas9-injected embryos to uninjected control, ns, *P* = 0.8406. (**I**) Single confocal plane of whole-mount embryos showing skeletal muscle cells and their nuclei stained with anti-myosin heavy chain antibody (mf20) and DAPI, respectively. Myosin assembles within the sarcomeres in controls, while it forms aggregates in *ninj1*-deficient embryos (in insets, apical section). Somites do not display the typical chevron shape, red line. Scale bar, 20 μm. (**J**) Somite length was significantly shorter in *ninj1*-deficient embryos compared to controls; **, *P* = 0.0025.

To assess the role of Ninjurin1 *in vivo*, we turned to the well-established zebrafish model. We used either CRISPR/Cas9 genome editing [31, 32] to create targeted mutations in the *ninjurin1* gene or morpholino oligonucleotide-mediated knockdown to assess the functional role of Ninjurin1 in muscle development. While uninjected and Cas9-control injected embryos displayed a normal morphology, the loss of *ninjurin1* induced by either *ninjurin1* sgRNA/Cas9 somatic mutagenesis (*ninj1* sgRNA) or by morpholino-mediated knockdown (*ninj1*^*e2i2*^ MO) lead to body axis and cardiac defects manifested as shorter body length and cardiac edema, respectively (Fig 7A and 7B), indicating impaired heart and skeletal muscle development. To validate the gene-specific effects of both knock-down technologies, we amplified the splicing products formed in uninjected, control morpholino-injected (control MO), and *ninj1*^*e2i2*^ MO-injected embryos. Our results showed splicing defects specific to *ninj1*^*e2i2*^ MO (Fig 7C). DNA sequencing of the *ninjurin1* sgRNA targeted region of the *ninjurin1* gene revealed various mutations at the cut site, with deletions ranging from two to twenty-two base pairs (Fig 7D). qRT-PCR showed a significant reduction in *ninjurin1* expression in *ninj1* sgRNA-Cas9-injected but not in Cas9-injected embryos (Fig 7E). To assess the heart morphology in more detail, we reduced *ninjurin1* levels in the transgenic zebrafish reporter line *Tg(myl7*:*EGFP)*^twu34^ expressing enhanced green fluorescent protein (EGFP) specifically in cardiomyocytes [33]. The most significant findings we observed were defects in cardiac looping with increased frequency of right sided hearts, so called dextrocardia (Fig 7F and 7G), whereas heart rate was not affected (Fig 7H). The profound body axis defects prompted us to examine skeletal muscle morphology. The skeletal muscle cells in *ninj1*^*e2i2*^ MO embryos formed shorter somites, which was associated with the loss of the typical chevron shape (Fig 7I and 7J). Moreover, the myosin heavy chain protein that normally assembles within the sarcomeres appeared aggregated, especially in the apical regions of the myocytes in *ninj1*^*e2i2*^ MO embryos when compared to uninjected controls (Fig 7I, insets). All together, these phenotypes indicate that Ninjurin1 plays an important role in heart and skeletal muscle development in zebrafish embryos.

## Discussion

We identified Ninjurin1 as potential regulator of myocyte hypertrophy and myogenic differentiation. Ninjurin1 expression was increased in pathological cardiac hypertrophy of man and mice *in vivo* and cardiomyocytes *in vitro*. Knockdown of Ninjurin1 inhibited agonist-induced cardiomyocyte hypertrophy. Also, deletion of Ninjurin1 led to defects in cardiac looping, dextrocardia, and skeletal muscle formation in zebrafish. These cardiac development and myogenesis phenotypes could be related to Ninjurin1`s effects on myogenic differentiation, which was increased by Ninjurin1 overexpression and diminished by its knockdown. In muscle, Ninjurin1 occurred as a 16kDa (Ninjurin1-16) and a 24kDa (Ninjurin1-24) variant. While myoblasts only contained Ninjurin1-16 which was associated with stress fibers, differentiated myotubes additionally contained the *N*-glycosylated Ninjurin1-24 variant regulating hypertrophy and myogenesis. Ninjurin1-16 and Ninjurin1-24 showed distinct subcellular localizations suggesting that Ninjurin1 depending on its modification has additional functions aside from its well described role as cell surface and adhesion molecule. Collectively our results suggest that Ninjurin1 plays an important role in myocyte growth and differentiation.

### Cardiac hypertrophy requires an increase in cell-cell and cell-matrix contacts

Within the myocardium of the adult heart cardiomyocytes physically interact with each other as well as with non-cardiomyocytes to form an intact syncytium. During pathological cardiac hypertrophy the composition of the myocardium is dramatically altered by cardiomyocyte growth [34], fibroblast activation, deposition of extracellular matrix proteins, and neovascularization [35]. To nevertheless assure a proper mechanical and electro-mechanical coupling and to withstand increased cardiac workload, hypertrophied myocytes have to be functionally embedded in the myocardial syncytium [36]. Therefore cell-cell and cell-matrix interactions, mediated by cell surface proteins, increase [37]. We found that Ninjurin1 was expressed in cardiomyocytes and upregulated during pathological cardiac hypertrophy in both patients and mice. Only few cell adhesion molecules, such as β-1D integrin [38], neural cell adhesion molecule (NCAM) [6], and intercellular adhesion molecule-1 (ICAM-1) [5] are involved in cardiac hypertrophy. For example, integrins are membrane-spanning proteins that connect the sarcomere to the extracellular matrix and regulate intracellular signaling pathways. Overexpression of integrin β-1D, a muscle-specific isoform, augments the hypertrophic response induced by α1 adrenergic stimulation in neonatal cardiomyocytes *in vitro* [38]. Importantly, integrins activate Akt and mitogen-activated protein kinase signaling pathways, which control the hypertrophic response of cardiomyocytes [39]. Like integrins, Ninjurin1 was also described as cell-surface protein mediating cell-cell interactions [7] and could thus be involved in myocyte growth and mediate pathological cardiac hypertrophy. Thus far, no data about Ninjurin1`s mediated signaling processes in myocytes are available. However, our finding that loss on Ninjurin1 reduces the hypertrophic response of NRVM and H9c2 cells indicates that Ninjurin1 is involved in hypertrophic signaling *in vitro*. In endothelial cells, Ninjurin1 was demonstrated to modulate p38 and activator protein 1 activity [40], and p53-dependent cell proliferation and apoptosis *in vitro* and *in vivo* [41]. Further studies are needed to investigate if Ninjurin1 activates one of these or other signaling pathways in myocytes.

We found that in myocardium, Ninjurin1 expression was low at baseline but strongly increased in cardiac hypertrophy. Interestingly, basal expression levels of Ninjurin1 in brain and neurons were also low, but increased during development, inflammation and injury [7]. Given the prominent role of Ninjurin1 in inflammation [40] and nerve repair [7, 13, 14], transcriptional regulation of Ninjurin1 seems to be important for its function. As Ninjurin1, also NCAM increases in the myocardium after heart transplantation [6] and ICAM increases during enhanced work load [5]. Collectively, reported and our own data indicate that increased Ninjurin1 expression is a prerequisite for its function in myocyte hypertrophy.

### Role of Ninjurin1 for cardiac development

We observed inhibition of cardiac looping and dextrocardia in Ninjurin1 mutant zebrafish. To our knowledge, this observation is the first *in vivo* demonstration of a functional significance of Ninjurin1 during cardiogenesis. The crucial event of cardiac looping is tightly regulated and requires multiple factors [42]. The knowledge of these factors is incomplete, but molecules involved in cell adhesion are important, such as NCAM [6] that regulates cardiac morphogenesis. Interestingly, FISH analysis localized the human *NINJURIN1* gene to chromosome 9q22.1-q22.3 [7]. A linkage analysis of patients with familial dilated cardiomyopathy identified a putative disease locus at chromosome 9q13-q22 [43]. Together with our results, it is possible that *Ninjurin1* is a disease gene for dilated cardiomyopathy. Nevertheless, the precise role of Ninjurin1 in cardiac morphogenesis needs further investigation.

### Role of Ninjurin1 in myogenesis

We found that Ninjurin1 protein was expressed as a Ninjurin1-16 and Ninjurin1-24 variant. Both forms were differentially expressed in a cell, tissue and condition dependent manner. By using tunicamycin-induced inhibition we showed that Ninjurin1 was *N*-glycosylated (Ninjurin1-24). *N*-glycosylation is a major post-translational modification of proteins in eukaryotic cells that influences the physicochemical and biological properties of proteins, such as protein stability and dynamics, as well as cell–matrix and cell–cell interactions [44, 45]. The importance of *N*-glycosylation is demonstrated by the fact that its complete absence is embryonically lethal [46]. In addition, genetic defects that affect protein glycosylation are associated with various human diseases [47]. Since Ninjurin1 was described as a cell surface protein, our observation is in line with the notion that *N*-linked glycosylation is a hallmark of cell membrane and extracellular proteins [20]. For example, *N*-glycosylation influences the subcellular localization and stability of various cell adhesion molecules, such as E- and N-cadherin [48] and integrins [49] where it affects intercellular signal transduction and cell–cell adhesion. In fact, *N*-glycosylation of Ninjurin1 was recently described by Bae et al. to be important for its oligomerization [15]. To these data, we add that *N*-glycosylation of Ninjurin1 in myocytes affects its stability and its subcellular localization. Our results that *N*-glycosylation of Ninjurin1 (Ninjurin1-24) primarily occurs during myogenic differentiation and myocyte and cardiac hypertrophy, and decreases during myocyte atrophy together with the observation of Bae et al. implicate that *N*-glycosylation of Ninjurin1 is important for myocyte growth and muscular adaptation during pathological stress. Our findings that Ninjurin1-24 has a shorter half-life in cycloheximide chase experiments and is primarily targeted by siRNA treatment when compared to Ninjurin1-16 suggests that *N*-glycosylation of Ninjurin1 reduces its stability, which is in line with the effects of *N*-glycosylation for other membrane proteins, such as E- and N-cadherin and integrins [48, 49]. Cell-cell adhesion is a prerequisite for myogenic differentiation. Absence of myogenic differentiation in myoblasts devoid of *N*-glycosylated Ninjurin1 argues for its function in this process. This was further supported by our result that Ninjurin1-24 was exclusively detected in differentiating myocytes, increased during hypertrophy and decreased during atrophy; whereas Ninjurin1-16 was continuously expressed in myoblasts, and differentiating and atrophying myotubes. The hypothesis that Ninjurin1 participates in myogenic differentiation was also supported by our observation that reduction of Ninjurin1 decreased and overexpression of Ninjurin1 increased the expression of the contractile protein myosin heavy chain, which is essential for myocyte development, growth and function, and used as a marker for myogenic differentiation [30]. Since Ninjurin1 specific siRNA abolished myogenic differentiation and predominantly reduced Ninjurin1-24 but not Ninjurin1-16 we believe that myogenic differentiation is primarily regulated by *N*-glycosylated Ninjurin1 (Ninjurin1-24).

Ninjurin1 was described as cell surface protein in non-myocytes [7, 8]. We show that in myocytes, Ninjurin1 was not exclusively localized to the cell surface but also in the cytoplasm. With fractionation assays we separated both Ninjurin1 variants; Ninjurin1-16 was primarily found in the cytoplasm whereas Ninjurin1-24, as it was reported for other glycoproteins, distributed to membranous fractions. In myoblasts Ninjurin1-16 co-localized with α-actinin, which is involved in assembly of myofibers and localizes to cell extensions and focal adhesions in C2C12 myoblasts [50]. In summary, our data indicate that Ninjurin1-16 is possibly involved in migration and fusion of myocytes while Ninjurin1-24 mainly executes more myocyte specific functions, such as mediating myogenic differentiation and myocyte growth.

## Conclusion

We conclude that Ninjurin1 is a multifunctional cell adhesion glycoprotein, which plays a role in myocyte hypertrophy and myogenesis. Depletion of Ninjurin1 in zebrafish impairs striated muscle development. To earlier observations that showed that Ninjurin1 is involved in nerve repair, inflammation and cancer progression, we add that Ninjurin1 has important functions during muscle homeostasis. Further analyses need to decipher specific signaling pathways downstream of Ninjurin1 in myocytes.

## Supporting information

S1 Fig Ninjurin1 is increased in hypertrophic left ventricle of patients. Western blots of proteins isolated from left ventricular tissue of patients with severe aortic stenosis (AS) undergoing elective aortic valve replacement surgery (*n* = 9) and donor hearts (*n* = 6) using anti-Ninjurin1 antibody. Tubulin and GAPDH were used as loading controls. The Ninjurin1 isoforms at 16kDa (Ninjurin1-16) and 24kDa (Ninjurin1-24) are differentially expressed therefore the membranes underwent short and long exposure, indicated by short exp. and long exp., respectively.
(EPS)

S2 Fig Ninjurin1-16 and Ninjurin1-24 distribute to distinct cytoplasmic fractions of myocytes. **(A)** Immunofluorescent staining of C2C12 myoblasts with anti-Ninjurin1 and anti-α-actinin as primary antibody, and Alexa Fluor 488 (green) and Alexa Fluor 555 (red) conjugated secondary antibody, respectively. Nuclei were stained with DAPI (blue). Scale bar, 75 μm. Inset (red box) is enlarged beside the panel. The arrow indicates stress fibers. **(B)** Immunofluorescent staining of C2C12 myoblasts and 5 days differentiated myotubes with anti-Ninjurin1 and anti-GAPDH as primary antibody, and Alexa Fluor 488 (green) and Alexa Fluor 555 (red) conjugated secondary antibody, respectively. Nuclei were stained with DAPI (blue). Scale bar, 75 μm. **(C)** C2C12 myoblasts and myotubes were subjected to biochemical cell fractionation. Proteins of each fraction were separated by SDS-PAGE and immunoblotted using anti-Ninjurin1, anti-AIF or anti- GAPDH antibody. The 16kDa (Ninjurin1-16) and 24kDa (Ninjurin1-24) Ninjurin1 isoforms are indicated. C indicates cytoplasmatic, M membranous, N nuclear, C1 cytosolic, M1 vesicular fractions, respectively.
(EPS)

S3 Fig Overexpression of Ninjurin1 accelerates differentiation of C2C12 myocytes. C2C12 myoblasts were transfected with cDNA expression plasmids encoding Flag-Ninjurin1 (n = 12) or empty vector control (Control) (n = 12). Differentiation was induced 24 hours post-transfection. Samples for RNA and protein isolation were obtained from myoblasts (0) and after 3, 5 and 7 days of differentiation as indicated. Three samples per group were analyzed (n = 3). (**A**) Western blots of proteins isolated from C2C12 myoblasts, and for 3, 5 and 7 days differentiated myotubes, as indicated (MT3, MT5, MT7), with anti-slow myosin, anti-fast myosin and anti-FLAG antibody (Flag-Ninjurin1) as indicated. GAPDH was used as loading control. A two-tailed, unpaired Student’s t-test was used to calculate the P values. (**B**) qRT-PCR analysis of myosin heavy chain (MyH) 1, 2, 4 and 7. *Glyceraldehyde-3 phosphate dehydrogenase* (*GAPDH*) expression was used as reference. Data are presented as mean ± SD. * P < 0.05, ** P < 0.01, § P < 0.001 compared to myoblast control.
(EPS)

S1 Table Clinical and echocardiographic data of patients with severe aortic stenosis (AS) undergoing elective aortic valve replacement surgery and donors. (DOCX)

S2 Table Morphological and echocardiographic parameters two weeks after sham and transverse aortic constriction (TAC) surgery. (DOCX)

S3 Table Morphological parameters 2 weeks after Angiotensin II (Ang II) and vehicle treatment. (DOCX)

S4 Table Primer pairs for CRISPR/Cas9 in zebrafish are shown. (DOCX)

S5 Table Primer pairs for real time RT-PCR are shown. (DOCX)

S1 File Supplementary Material and Methods. (DOCX)
