# Supplementary material for: Ninjurin1 regulates striated muscle growth and differentiation
Source: PLoS One. 2019 May 15;14(5):e0216987. doi: 10.1371/journal.pone.0216987 (PMC6519837; doi:10.1371/journal.pone.0216987)
Supplement: S1 Table — (DOCX) [file pone.0216987.s004.docx]

**S1 Table.** Clinical and echocardiographic data of patients with severe aortic stenosis (AS) undergoing elective aortic valve replacement surgery and donors.

|  | **AS**  (*n* = 9) | **Donor**  (*n* = 6) |
| --- | --- | --- |
| Age at surgery, years | 63.3±3.1 | 53.8±0.7 |
| Women (n, %) | 3 (30) | 3 (50) |
| BSA, m^2^ | 2.01±0.07 | n.d. |
| NYHA functional class II/III (%) | 77.8 | n.d. |
| **LV morphology in echocardiography** | |  |
| LVID_(d)_, mm | 49.7±2.2 | n.d. |
| LVID_(s)_, mm | 32.5±2.3 | n.d. |
| PWT_(d)_, mm | 12.0±0.31 | n.d. |
| IVS_(d)_, mm | 12.7±0.50 | n.d. |
| LVEF, % | 59.44±1.46 | n.d. |
| P_mean_, mmHg | 58.78±4.06 | n.d. |
| P_peak_, mmHg | 77.67±7.77 | n.d. |

Values are mean ± standard deviation (SD). BSA indicates body surface area; IVS_(d)_, end-diastolic interventricular septum thickness; LV, left ventricular; LVEF, left ventricular ejection fraction; LVID, left ventricular internal dimension at end-diastole (d) and end-systole (s); NYHA, New York Heart Association; P_mean_, mean pressure gradient; P_peak_, peak pressure gradient; PWT_(d)_, end-diastolic posterior wall thickness. n.d., not determined.
