# Supplementary material for: Ninjurin1 regulates striated muscle growth and differentiation
Source: PLoS One. 2019 May 15;14(5):e0216987. doi: 10.1371/journal.pone.0216987 (PMC6519837; doi:10.1371/journal.pone.0216987)
Supplement: S2 Table — (DOCX) [file pone.0216987.s005.docx]

**S2 Table.** Morphological and echocardiographic parameters two weeks after sham and transverse aortic constriction (TAC) surgery.

|  | **sham**  (*n* = 4) | **TAC**  (*n* = 8) | ***P* value** |
| --- | --- | --- | --- |
| **Morphological parameter** | |  |  |
| Body weight, g | 26.05±2.56 | 25.26±0.72 | 0.535 |
| HR, beats/min | 439±21 | 456±19 | 0.865 |
| HW, mg | 115±16 | 160±23 | 0.009 |
| HW/TL, mg/mm | 6.71±0.88 | 9.26±1.16 | 0.005 |
| LW/TL, mg/mm | 11.8±1.5 | 12.6±2.2 | 0.541 |
| **Echocardiography** | |  |  |
| TPG, mmHg |  | 54.11±2.91 |  |
| LVEF, % | 40.23±2.77 | 48.72±8.97 | 0.102 |
| LVID_(d)_, mm | 4.36±0.27 | 3.96±0.27 | 0.052 |
| LVID_(s)_, mm | 3.51±0.15 | 3.00±0.35 | 0.029 |
| IVS_(d)_, mm | 0.72±0.09 | 1.21±0.16 | 0.001 |
| PW_(d)_, mm | 0.59±0.03 | 1.09±0.15 | 0.001 |

Values are mean ± standard deviation (SD). Measurements were performed after two weeks of sham and TAC surgery, respectively. HW indicates heart weight; HW/TL, HW/tibia length; LVID, LV internal diameter during diastole (d) and systole (s); PW_(d)_, posterior wall thickness during diastole; IVS_(d)_, thickness of the interventricular septum during diastole; LVEF, left ventricular ejection fraction; TPG, transaortic pressure gradient; LW, lung weight; HR, heart rate.
