# Supplementary material for: Ninjurin1 regulates striated muscle growth and differentiation
Source: PLoS One. 2019 May 15;14(5):e0216987. doi: 10.1371/journal.pone.0216987 (PMC6519837; doi:10.1371/journal.pone.0216987)
Supplement: S3 Table — (DOCX) [file pone.0216987.s006.docx]

**S3 Table.** Morphological parameters 2 weeks after Angiotensin II (Ang II) and vehicle treatment.

|  | **vehicle**  (*n* = 5) | **Ang II**  (*n* = 5) | ***P* value** |
| --- | --- | --- | --- |
| Body weight, g | 29.9±2.5 | 24.6±3.1 | 0.004 |
| HR, beats per minute | 455±28 | 463±32 | 0.846 |
| HW, mg | 134.4±9.6 | 162.1±33.8 | 0.048 |
| HW/TL, mg/mm | 7.52±0.54 | 9.15±1.7 | 0.03 |
| LW/TL, mg/mm | 8.8±1.0 | 9.9±3.4 | 0.423 |

Values are mean ± standard deviation (SD). Measurements were performed after 2 weeks of vehicle and Ang II treatment, respectively. HW indicates heart weight; HW/TL, HW/tibia length; LW, lung weight; HR, heart rate.
