# Supplementary material for: Ninjurin1 regulates striated muscle growth and differentiation
Source: PLoS One. 2019 May 15;14(5):e0216987. doi: 10.1371/journal.pone.0216987 (PMC6519837; doi:10.1371/journal.pone.0216987)
Supplement: S4 Table — (DOCX) [file pone.0216987.s007.docx]

**S4 Table.** Primer pairs for CRISPR/Cas9 in zebrafish are shown. Splice blocking together with standard control morpholino oligo are shown together with primers for detection of splice-blocked variant.

| **Gene** | **Primer sequence** |
| --- | --- |
| Dr_*ninj1*_flanking-exon1_forward | 5'-GAAAGATCCGACTCTCAAAGAACG-3' |
| Dr_*ninj1*_flanking-exon1_reverse | 5'-CCGCAGCTTATCCGTGAAATAC-3' |
| Dr_CRISPR- exon1_Fw | 5'-GAAATTAATACGACTCACTATAGGAATGGAGGTG  TAAACCGAGTTTTAGAGCTAGAAATAGC-3' |
| Dr_sgRNA_R | 5'-AAAAGCACCGACTCGGTGCCACTTTTTCAAGTT  GATAACGGACTAGCCTTATTTTAACTTGCTATTTCTAGCTCTAAAAC-3' |
| MO-*ninj1*-e2i2 | 5'-AGGAGAGCTGGTCTCACCTATGAA-3' |
| MO-standard control | 5'-CCTCTTACCTCAGTTACAATTTATA-3' |
| Dr_*ninj1*_exon2_forward | 5'-CAACAAGAAGAGTGCAGCGGA-3' |
| Dr_*ninj1*_exon3_reverse | 5'-ACGATGAAGACGAGAGCGGTG-3' |

*ninj1* indicates Ninjurin1; Dr, Danio rerio.
