# Supplementary material for: Ninjurin1 regulates striated muscle growth and differentiation
Source: PLoS One. 2019 May 15;14(5):e0216987. doi: 10.1371/journal.pone.0216987 (PMC6519837; doi:10.1371/journal.pone.0216987)
Supplement: S5 Table — (DOCX) [file pone.0216987.s008.docx]

**S5 Table.** Primer pairs for real time RT-PCR are shown.

| **Gene** | **Primer sequence** |
| --- | --- |
| Mm_*Gapdh*_forward | 5'-ATG GTG AAG GTC GGT GTG A-3' |
| Mm_*Gapdh*_reverse | 5'-AAT CTC CAC TTT GCC ACT GC-3' |
| Mm_*Ninj1*_forward | 5'-TCG TGC TGC AGA TAG GAG TG-3' |
| Mm_*Ninj1*_reverse | 5'-GAA GAT GTT GAC CAC GAC GA-3' |
| Mm_*Myh1*_forward | 5'-AAT CAA AGG TCA AGG CCT ACA A-3' |
| Mm_*Myh1*_reverse | 5'-GAA TTT GGC CAG GTT GAC AT-3' |
| Mm_*Myh2*_forward | 5'-AAC TCC AGG CAA AAG TGA AAT C-3' |
| Mm_*Myh2*_reverse | 5'-TGG ATA GAT TTG TGT TGG ATT GTT-3' |
| Mm_*Myh4*_forward | 5'-TGG CCG AGC AAG AGC TAC-3' |
| Mm_*Myh4*_reverse | 5'-TTG ATG AGG CTG GTG TTC TG-3' |
| Mm_*Myh7*_forward | 5'-CGC ATC AAG GAG CTC ACC-3' |
| Mm_*Myh7*_reverse | 5'-CTG CAG CCG CAG TAG GTT-3' |
| Mm_*Nppa*_forward | 5'-GGG GGT AGG ATT GAC AGG AT-3' |
| Mm_*Nppa*_reverse | 5'-ACA CAC CAC AAG GGC TTA GG-3' |
| Mm_*Nppb*_forward | 5'-GCA CAA GAT AGA CCG GAT CG-3' |
| Mm_*Nppb*_reverse | 5'-CTT CAA AGG TGG TCC CAG AG-3' |
| Rn_*Gapdh*_forward | 5'-CAA GGT CAT CCA TGA CAA CTT TG-3' |
| Rn_*Gapdh*_reverse | 5'-GGG CCA TCC ACA GTC TTC TG-3' |
| Rn_*Myh6*_forward | 5'-TGCAGAAGAAACTGAAGGAAA-3' |
| Rn_*Myh6*_reverse | 5'-GCT CCG CCT CTA GCT CCT-3' |
| Rn_*Nppb*_forward | 5'-TCC AAG ATG GCA CAT AGT TCA-3' |
| Rn_*Nppb*_reverse | 5'-AGC CCA AGC GAC TGA CTG-3' |
| Rn_*18S rRNA*_forward | 5'-GCA ATT ATT CCC CAT GAA CG-3' |
| Rn_*18S rRNA*_reverse | 5'-GGG ACT TAA TCA ACG CAA GC-3' |
| Rn_*Ninj1*_forward | 5'-GAA GCC TGT CAT GGA CGT G-3' |
| Rn_*Ninj1*_reverse | 5'-GAT GCC TTG GGG ATA CAG C-3' |

Ninj1 indicates Ninjurin1; Nppa, natriuretic peptide A; Nppb, B-type natriuretic factor; Myh, myosin heavy chain; Mm, Mus musculus; Rn, Rattus norvegicus.
