# Supplementary material for: Ninjurin1 regulates striated muscle growth and differentiation
Source: PLoS One. 2019 May 15;14(5):e0216987. doi: 10.1371/journal.pone.0216987 (PMC6519837; doi:10.1371/journal.pone.0216987)
Supplement: S1 File — (DOCX) [file pone.0216987.s009.docx]

**S1 File. Supplementary Material and Methods**

**Zebrafish strains, maintenance and analyses**

Zebrafish were maintained in accordance with the guidelines of the Max-Delbrück Center for Molecular Medicine and the local authority for animal protection (Landesamt für Gesundheit und Soziales, (LaGeSo) Berlin, Germany) for the use of laboratory animals, and followed the ‘Principles of Laboratory Animal Care’ (NIH publication no. 86-23, revised 1985) as well as the current version of German Law on the Protection of Animals. Zebrafish embryos were raised in E3 embryo medium (130 mM NaCl, 0.5 mM KCl, 0.02 mM Na_2_HPO_4_, 0.04 mM KH_2_PO_4_, 1.3 mM CaCl_2_, 1.0 mM MgSO_4_, and 0.4 mM NaH_2_CO_3_) [1] containing N-phenylthiourea (PTU; Sigma-Aldrich) and kept under standard laboratory conditions at 28.5 °C. Transgenic line *Tg(myl7:EGFP)*^twu34^ [2], which expresses enhanced green fluorescent protein (EGFP) in cardiomyocytes, and AB/Tuebingen wild type line were used in this study.

**CRISPR/Cas9: sgRNA design, production, and microinjection**

The CRISPR design tool from the Zhang lab (MIT 2013, http://crispr.mit.edu/) was used to search for sgRNA target sites corresponding to 18 NGG on the sense or antisense strand of the zebrafish Ninjurin1 DNA. The basic local alignment search tool (BLAST) applied to the zebrafish genome GRCz10 was used to exclude putative off-target binding of designed oligonucleotides. PCR was performed with Phusion High-Fidelity DNA Polymerase (Thermo Fisher Scientific Inc.) in High-Fidelity buffer with oligonucleotides CRISPRF (a unique oligonucleotide encoding the T7 polymerase binding site and the sgRNA target sequence in exon 1 of Ninjurin1) and sgRNAR (a common oligonucleotide encoding the remainder of the sgRNA sequence) [3, 4] in 100 µl reaction volume (for primer sequences please refer to S4 Table). The MEGAscript® T7 Kit (Ambion® GmbH) was used for in vitro transcription of 1000 ng purified DNA template at 37°C overnight. sgRNA was purified by RNeasy® Mini Kit (QIAGEN®). sgRNAs were diluted in Diethylpyrocarbonat (DEPC)-treated water to 600 ng/µl and stored in aliquots at -80 °C until usage. Prior to injection a solution containing 120 ng/µl sgRNA and 120 ng/µl recombinant Cas9 protein, both endotoxin-free, was prepared as previously reported [5, 6]. Single-cell stage embryos were injected with 1 nl of this injection solution per embryo. Embryos were incubated at 28.5 °C for indicated time points.

**Extraction and sequence analysis of genomic DNA from zebrafish**

Genomic DNA was extracted from single zebrafish embryos (48 hpf) by homogenization in 50 µl of 50 mM NaOH and heating to 95 °C for 10 min. 5 µl 1.0 M Tris-HCl (pH 8.2) was added to the genomic DNA extract, and the mixture was stored at 4°C. Single embryo genomic DNA, primer (for primer sequences please refer to S4 Table) and Pfu polymerase were used to amplify the flanking sequence (±500 bp) around the sgRNA targeted genomic region. The amplicon was cloned into the pGEM®-T Easy Vector system (Promega GmbH) and Sanger sequencing was performed. At least 10 sequences per embryo were analysed with CrispRVariants to assess the frequency of individual alleles [7]

**Morpholino-mediated knockdown**

The morpholinos used were obtained from GeneTools, LLC (for sequences please refer to S4 Table). The *MO-ninj1* targets the 3’ end of the exon-intron junction of exon2. Single-cell stage embryos were injected with approximately 1,7 pg of morpholino per embryo. Embryos were incubated at 28.5 °C for indicated time points.

**RT-PCR and qPCR**

Total RNA was isolated from 50 hpf zebrafish embryos using TRIzol® Reagent (Invitrogen™, #15596026). On-column DNase I treatment (Appendix D: DNase Digestion of RNA before RNA Cleanup), clean-up and concentration of RNA were performed according to the RNeasy Mini Kit (Qiagen, #74104). mRNA was transcribed into cDNA using First-Strand cDNA Synthesis Kit (ThermoFisher Scientific™, #K1612) according to manufacturer’s protocol with combined random hexamer and oligo(dT) primed synthesis (1:2). 100 ng of the reverse transcription reaction product was immediately used for RT-PCR (for primer sequences please refer to S4 Table) or qPCR with FAM™ dye-labeled TaqMan® MGB probes and TaqMan™ Gene Expression Master Mix (Applied Biosystems™, #10525395) on a ViiA™ 7 Real-Time PCR System. The qPCR reaction was performed in technical triplicates including minus-reverse transcriptase (–RT) and no-template controls. qPCRs were performed for 50 cycles. *Eef1α1l* labelled with VIC™ dye was used as internal reference gene and pipetted in the same well as genes of interest. All C_T_ values were normalized to that of the reference gene in the same well, fold induction (fold change, FC) was calculated using the ΔΔC_T_ method and plotted as log_2_(FC). Statistics were done with GraphPad Prism 7 on the ΔC_T_ values comparing the untreated control situation to the treated situation using unpaired t-test with Welch correction.

**Whole mount immunochemistry and microscopy of zebrafish embryos**

Immunostaining was carried out using 48 hpf whole mount zebrafish embryos. Zebrafish embryos were fixed in PEM buffer (0.1 M PIPES (pH 6.95), 1 mM MgSO_4_, 2 mM EGTA in DEPC water) containing 4% Formaldehyde and 0.1% Triton X-100 for 2 hours at room temperature. The fixed embryos were washed 3 times for 15 minutes in Blocking buffer (0.1% bovine serum albumin, 1% dimethylsulfoxid, 5% goat serum, 0.1% Triton X-100 in PBS). Whole-mount immunohistochemistry was performed with mouse-anti-myosin (Developmental Studies Hybridoma Bank; MF 20-s; 1:200) as primary antibody, and donkey-anti-mouse-Alexa® Fluor 488 conjugated antibody (Invitrogen / Life technologies, 1:1000) as secondary antibody. A Leica TCS SP8-Confocal Laser Scanning microscope equipped with an argon-laser was used to obtain confocal images. A Leica M80 stereomicroscope was used for capturing bright field and fluorescent images. *Heart rate and heart looping measurements.* Heart rate was measured manually in 48 hpf old embryos. For each condition, 30 embryos were used. To measure the angle of cardiac looping, embryos were embedded in 1.5 % methylcellulose (Sigma) dissolved in E3 embryo medium, and imaged with the Leica M80 stereomicroscope. The looping angle was defined as an angle between the plane of AVJ and the embryo midline axis as previously described [8]. For somite length, confocal images were used to quantify at least 3 somites per embryo at 6 random positions, and averaged per embryo. Images were analysed using ImageJ/Fiji [9]. Statistical analysis was performed using GraphPad Prism®. All zebrafish data are reported as the sample mean ± standard deviation (SD). Statistical significance of heart looping angle was tested with a two tailed, unpaired Student t-test, *P* = 0.0353. Differences in heart rate were analysed by one-way ANOVA.

**BD Pharmingen PowerBlot^TM^**

Myocardial samples of the left ventricular part of the interventricular septum from patients with aortic stenosis (AS, *n* = 3) and, for logistical reasons not used, donor hearts (controls, *n* = 3) were obtained during cardiac surgery and organ harvest, respectively. Recently we reported on these patients elsewhere [10]. Proteins were extracted from tissue samples according to the manufacturers’ protocol and analysed by the BD PowerBlot^TM^ Western Array Screening Service. Shortly a set of 1000 monoclonal antibody was used in a high throughput Western blot screening to detect differentially expressed proteins in AS compared to controls. Densitometric measurements of immunoreactive bands and normalization of signals were performed by BD Pharmingen^TM^.

**Cell culture**

C2C12 and H9c2 myoblasts were grown in Dulbecco's Modified Eagle's medium (DMEM; 1g/L Glucose) with 10 % fetal bovine serum, 2 mM glutamine and 1 U/ml penicillin, 1 µg/ml streptomycin at 37°C in a 5% CO_2_ atmosphere. For differentiation, growth medium was replaced at confluence of cells by medium containing 2% FBS for C2C12 and 1% FBS for H9c2, respectively. Differentiated C2C12 and H9c2 myotubes were exposed to dexamethasone (Dexa, 10 µM) to induce atrophy. Ethanol (99.6 % v/v) was used as the accordant solvent control. Differentiated H9c2 myotubes (MT7) were exposed to arginine-vasopressin (AVP, 100 nM) for 24 hours to induce hypertrophy. Water was used as solvent control for these compounds. To inhibit glycosylation, differentiating H9c2 cells were incubated with tunicamycin (1 µg/mL) for 24 h. Dimethyl sulfoxide (DMSO) was used as the accordant solvent control. CHQ myoblasts were treated as recently described [11]. Shortly, CHQ cells were cultivated in growth medium (4 volumes of DMEM, 1g/L glucose) plus 1 volume of M199 containing 20% FBS and 50 µg/ml gentamycin). For differentiation, growth medium was replaced by differentiation medium (DMEM, 1 g/L glucose), 50 µg/ml gentamycin) at confluence of cells. Other cells were maintained in (DMEM, 4.5 g/l glucose) containing 10 % fetal bovine serum, 2 mM glutamine and 1 U/ml penicillin, 1 µg/ml streptomycin.

**In vitro cardiomyocyte experiments**

Neonatal rat cardiomyocytes (NRVM) were isolated from 1 to 3 days old Wistar rats as described previously [12]. After decapitation, hearts were removed, atria excised and ventricles were minced and collected in 1xPBS. Ventricular tissue was digested using digestion buffer (1xPBS, 0.5 mg/ml collagenase type II (Worthington), 0.6 mg/ml pancreatin (Sigma Aldrich)) and spun in a spinner bottle at low speed. After 20 min the digestion buffer containing cardiomyocytes and other cell types were removed, centrifuged (3 min, 1200 rpm, RT) and resuspended in plating medium (DMEM containing 10 % FBS (Biochrom), 1 % L-glutamine (Sigma Aldrich) and 1 % penicillin/streptomycin (Sigma Aldrich)). New digestion buffer was then added to the ventricular tissue and the procedure was repeated six times. The collected cell suspensions were pooled, filtered through a 70 µm Nylon cell strainer (BD) and contaminating fibroblasts were removed by pre-plating for 1 hour as described. After 36-48 hours NRVM were set to serum-free medium for 24 hours and then treated with phenylephrine (100 µM, Sigma Aldrich) or endothelin 1 (50 nM, Sigma Aldrich) for 24 hours to induce hypertrophy. For siRNA transfection 1 to 1.5 x 10^6^ cells per well on a six well plate were plated using growth medium without antibiotics and incubated at 37°C in a 5% CO_2_ atmosphere for 24 hours. 200 µl of transfection mixture per well consisting of 5 µl of siRNA (100 nM, Dharmacon / Thermo Scientific), 5 µl of Dharmafect3 (Dharmacon/Thermo Scientific) and 190 µl of serum-free medium (DMEM, containing 1% glucose) was prepared and incubated at room temperature for 20 minutes according to the manufacturers protocol. Following 24 hours of transfection cells were treated with 100 µM phenylephrine (Sigma Aldrich), 50 nM endothelin 1 or vehicle (1xPBS) as indicated for additional 24 hours. RNA isolation, cDNA synthesis and quantitative real-time RT-PCR were performed as recently described [13-16].

**Transfection of cDNA expression plasmids and siRNA in cells**

For transfection of plasmids 75,000 C2C12 cells per well on a six well plate were seeded using growth medium (DMEM with low glucose , 1 g/L), supplemented with 10 % Fetal bovine serum (FBS), 2 mM glutamine) without antibiotics and incubated at 37°C in a 5% CO_2_ atmosphere for 18 h. 200 µl of transfection mixture per well consisting of 6 µl of Polyethyleneimin (4 mg/ml) and 2µl of plasmid DNA (1µg/µl) in Opti-MEM^®^ (Life Technologies) were prepared and incubated at room temperature for 20 minutes according to the manufacturers protocol. For siRNA transfection of C2C12 cells 100,000 cells per well on a six well plate were plated using growth medium without antibiotics and incubated at 37°C in a 5% CO_2_ atmosphere for 18 hour. 400 µl of transfection mixture per well consisting of 10 µl of siRNA (100 nM, Dharmacon/Thermo Scientific), 10 µl of Dharmafect3 (Dharmacon/Thermo Scientific) and 380 µl of Opti-MEM^®^ (Life Technologies) were prepared and incubated at room temperature for 20 minutes according to the manufacturers protocol. Following 24 hour of transfection cells were either directly subjected to analysis or differentiated into myotubes. For siRNA transfection of H9c2 cells, 7 days differentiated myotubes were incubated with 100 nM siRNA for 48 hours before treatment with 100 nM arginine-vasopressin (AVP) for additional 24 hours.

**Cycloheximide chase assay**

To estimate half-life’s of endogenous proteins, 100,000 C2C12 cells per well were seeded on a six well plate and incubated at 37°C in a 5% CO_2_ atmosphere for 18 hours. Cycloheximide (CHX, 50 μg/ml) or water as control was added for indicated time points before the chase was stopped by cell lysis. In order to analyze proteasome dependent protein degradation the proteasome inhibitor MG132 (10 μM) was used as indicated.

**Protein extraction and Western blot analysis**

If not otherwise noted, tissues or cells were lysed in ice cold extraction buffer (50 mM Tris-HCl, 150 mM NaCl, 1 mM EDTA (sodium salt), 1% (v/v) NonidetP40, 0.5% (w/v) sodium deoxycholate, 0.1% (w/v) SDS and protease inhibitors (Complete^TM^, Roche), pH 8.0) as described recently [16]. Protein concentration of lysates was determined by measuring their absorbance at the wavelength of 280 nm in an UV-VIS spectrometer. Lysates were denatured using SDS sample buffer (50 mM Tris-HCl, 2 % SDS, 6 % glycerol, 5 % β-mercaptoethanol, 0.1 % bromophenol blue) and subjected to SDS-PAGE using 10 – 15 % acrylamide gels depending on protein size. Proteins were immunoblotted using the following primary antibodies: anti-Ninjurin1 (BD Biosciences), anti-DYKDDDDK_(Flag)-tag (Cell Signaling), anti-glyceraldehyde-3-phosphate dehydrogenase (GAPDH, Millipore), anti-Herpud1 (kindly provided by M. Seeger), anti-Myogenin (Clone F12B, Sigma), anti-Myosin (MHC, clone MF-20, R&D Systems), anti-Fast Myosin (clone My32, Sigma), anti-slow Myosin (clone NOQ7, Sigma), anti-tubulin (Cell Signaling), anti-gp130 (Abcam) and anti-Apoptosis inducing factor (AIF, Cell Signaling) antibody. GAPDH or tubulin signals were used as loading control, respectively. As secondary antibody we used the following anti-horseradish peroxidase (HRP) conjugated antibodies: anti-mouse IgG, anti-rabbit IgG (both Cell Signaling Technology). The signal was visualized with SuperSignal™ West Pico Chemiluminescent Substrate (Thermo Scientific). Densitometric analysis of Western blot signals was performed using Image J software (ImageJ 1.43u, http://rsb.info.nih.gov/ij).

**Biochemical fractionation of cells**

For biochemical fractionation cells were resuspended in buffer 1 (50 mM Tris pH 7.7; 50 mM NaCl, 5 mM MgCl_2_ and protease inhibitors (Complete^TM^)), snap frozen, subjected to three freeze and thaw cycles, and afterwards centrifuged at 1,000 x g for 10 minutes. The supernatant was declared as cytoplasm (**C**). The sediment was resuspended in buffer 2 (buffer 1 plus 1% NP-40), incubated on ice for 30 minutes and centrifuged at 6,000 x g for 10 minutes. The resulting sediment was declared as nuclei (**N**) and the supernatant as membranes (**M**). The C fraction was subjected to ultracentrifugation at 150,000 x g for 30 minutes. The sediment was declared as vesicular membranes (**M1**) and the supernatant as cytosol (**C1**). Equal amounts of each fraction were subjected to SDS PAGE and Western blot analysis. Purity of fractions was estimated by immunoblotting with antibodies against fraction specific proteins as indicated.

**RNA isolation, cDNA synthesis and qRT-PCR**

Total RNA was isolated from cells and tissue using TRIzol® reagent (Invitrogen / Life technologies) according to the manufacturer’s protocol. cDNA was synthesized using 1 µg of RNA and the SuperScript® First-Strand Synthesis kit (Invitrogen / Life technologies) according to the manufacturers protocol. Quantitative real time polymerase chain reaction (qPCR) was performed using SYBR Green PCR Master Mix (Roche) and self-designed primers (S5 Table). qPCR reactions were run in a Step-One^TM^ Plus thermocycler (Applied Biosystems) as described recently, using a cDNA standard curve [16]. Expression of glycerolaldehyde-3-phosphate dehydrogenase (*GAPDH*) was used as a reference.

**Immunostaining of cells and tissue samples**

NRVM, C2C12, H9c2, and CHQ cells were cultured on cover slips and fixed using 4 % paraformaldehyde in PBS. Frozen Sections of cardiac tissue were dried and fixed with acetone. Fixed tissues and cells were permeabilized with 0.2 % Triton-X-100 in PBS and blocked using 5 % goat serum. Anti-Ninjurin1 (BD, mouse monoclonal, 1:500 for C2C12, H9c2, and CHQ cells; 1:200 for NRVM), anti-GAPDH (Millipore, 1:5000), anti-α-Actinin (Sigma, 1:500), and anti-Ninjurin1 (Aviva, rabbit polyclonal, 1:500) antibody were used as primary antibody. As secondary antibodies, goat-anti-mouse-Alexa® Fluor 488, goat-anti-mouse-Alexa® Fluor 555, and goat-anti-rabbit-Alexa® Fluor 555 (Invitrogen / Life technologies, 1:1000) were used as indicated. Embedding was performed using ProLongGold® Antifade Reagent containing 4′,6-Diamidin-2-phenylindol (DAPI) for nuclear staining (Invitrogen / Life Technologies). Microscopy of C2C12, H9c2, and CHQ cells was performed using a Leica CTR 6500 fluorescence microscope combined with the Leica DFC 360 FX digital camera. Pictures from NRCM were taken using a Zeiss LSM 700 confocal microscope (Carl Zeiss MicroImaging GmbH).**Supplementary References**

1. Westerfield CR. A Guide for the Laboratory Use of Zebrafish. The Zebrafish Book 1995.

2. Huang CJ, Tu CT, Hsiao CD, Hsieh FJ, Tsai HJ. Germ-line transmission of a myocardium-specific GFP transgene reveals critical regulatory elements in the cardiac myosin light chain 2 promoter of zebrafish. Developmental dynamics : an official publication of the American Association of Anatomists. 2003;228(1):30-40. doi: 10.1002/dvdy.10356. PubMed PMID: 12950077.

3. Shah AN, Davey CF, Whitebirch AC, Miller AC, Moens CB. Rapid reverse genetic screening using CRISPR in zebrafish. Nature methods. 2015;12(6):535-40. doi: 10.1038/nmeth.3360. PubMed PMID: 25867848; PubMed Central PMCID: PMC4667794.

4. Bassett AR, Tibbit C, Ponting CP, Liu JL. Highly efficient targeted mutagenesis of Drosophila with the CRISPR/Cas9 system. Cell reports. 2013;4(1):220-8. doi: 10.1016/j.celrep.2013.06.020. PubMed PMID: 23827738; PubMed Central PMCID: PMC3714591.

5. Gagnon JA, Valen E, Thyme SB, Huang P, Akhmetova L, Pauli A, et al. Efficient mutagenesis by Cas9 protein-mediated oligonucleotide insertion and large-scale assessment of single-guide RNAs. PLoS One. 2014;9(5):e98186. doi: 10.1371/journal.pone.0098186. PubMed PMID: 24873830; PubMed Central PMCID: PMC4038517.

6. Burger A, Lindsay H, Felker A, Hess C, Anders C, Chiavacci E, et al. Maximizing mutagenesis with solubilized CRISPR-Cas9 ribonucleoprotein complexes. Development. 2016;143(11):2025-37. Epub 2016/05/01. doi: 10.1242/dev.134809. PubMed PMID: 27130213.

7. Lindsay H, Burger A, Biyong B, Felker A, Hess C, Zaugg J, et al. CrispRVariants charts the mutation spectrum of genome engineering experiments. Nat Biotechnol. 2016;34(7):701-2. Epub 2016/07/13. doi: 10.1038/nbt.3628. PubMed PMID: 27404876.

8. Merks AM, Swinarski M, Meyer AM, Muller NV, Ozcan I, Donat S, et al. Planar cell polarity signalling coordinates heart tube remodelling through tissue-scale polarisation of actomyosin activity. Nat Commun. 2018;9(1):2161. Epub 2018/06/06. doi: 10.1038/s41467-018-04566-1. PubMed PMID: 29867082; PubMed Central PMCID: PMCPMC5986786.

9. Schindelin J, Arganda-Carreras I, Frise E, Kaynig V, Longair M, Pietzsch T, et al. Fiji: an open-source platform for biological-image analysis. Nature methods. 2012;9(7):676-82. doi: 10.1038/nmeth.2019. PubMed PMID: 22743772; PubMed Central PMCID: PMC3855844.

10. Fielitz J, Dendorfer A, Pregla R, Ehler E, Zurbrugg HR, Bartunek J, et al. Neutral endopeptidase is activated in cardiomyocytes in human aortic valve stenosis and heart failure. Circulation. 2002;105(3):286-9. PubMed PMID: 11804980.

11. Langhans C, Weber-Carstens S, Schmidt F, Hamati J, Kny M, Zhu X, et al. Inflammation-induced acute phase response in skeletal muscle and critical illness myopathy. PLoS One. 2014;9(3):e92048. doi: 10.1371/journal.pone.0092048. PubMed PMID: 24651840; PubMed Central PMCID: PMC3961297.

12. Bush E, Fielitz J, Melvin L, Martinez-Arnold M, McKinsey TA, Plichta R, et al. A small molecular activator of cardiac hypertrophy uncovered in a chemical screen for modifiers of the calcineurin signaling pathway. Proc Natl Acad Sci U S A. 2004;101(9):2870-5. PubMed PMID: 14976250.

13. Du Bois P, Pablo Tortola C, Lodka D, Kny M, Schmidt F, Song K, et al. Angiotensin II Induces Skeletal Muscle Atrophy by Activating TFEB-Mediated MuRF1 Expression. Circ Res. 2015;117(5):424-36. doi: 10.1161/CIRCRESAHA.114.305393. PubMed PMID: 26137861; PubMed Central PMCID: PMC4537692.

14. Fielitz J, Kim MS, Shelton JM, Latif S, Spencer JA, Glass DJ, et al. Myosin accumulation and striated muscle myopathy result from the loss of muscle RING finger 1 and 3. J Clin Invest. 2007;117(9):2486-95. PubMed PMID: 17786241.

15. Lodka D, Pahuja A, Geers-Knörr C, Scheibe R, Nowak M, Hamati J, et al. Muscle RING-finger 2 and 3 maintain striated-muscle structure and function. . Journal of Cachexia, Sarcopenia and Muscle. 2015. doi: 10.1007/jcsm.12057.

16. Schmidt F, Kny M, Zhu X, Wollersheim T, Persicke K, Langhans C, et al. The E3 ubiquitin ligase TRIM62 and inflammation-induced skeletal muscle atrophy. Crit Care. 2014;18(5):545. doi: 10.1186/s13054-014-0545-6. PubMed PMID: 25263070; PubMed Central PMCID: PMC4231194.
